# MAPK Pathway under Chronic Copper Excess in Green Macroalgae (Chlorophyta): Involvement in the Regulation of Detoxification Mechanisms

**DOI:** 10.3390/ijms20184546

**Published:** 2019-09-13

**Authors:** Fernanda Rodríguez-Rojas, Paula S. M. Celis-Plá, Lorena Méndez, Fabiola Moenne, Pamela T. Muñoz, M. Gabriela Lobos, Patricia Díaz, José Luis Sánchez-Lizaso, Murray T. Brown, Alejandra Moenne, Claudio A. Sáez

**Affiliations:** 1 Laboratory of Aquatic Environmental Research, Centro de Estudios Avanzados, Universidad de Playa Ancha, Viña del Mar 2520000, Chile; 2 HUB-AMBIENTAL UPLA, Universidad de Playa Ancha, Valparaíso 2340000, Chile; 3 Doctorado Interdisciplinario en Ciencias Ambientales, Facultad de Ciencias Naturales y Exactas, Universidad de Playa Ancha, Valparaíso 2340000, Chile; 4 Doctorado en Ciencias del Mar y Biología Aplicada, Departamento de Ciencias del Mar y Biología Aplicada, Universidad de Alicante, 03080 Alicante, Spain; 5 Laboratory of Environmental and Analytical Chemistry, Instituto de Química y Bioquímica, Facultad de Ciencias, Universidad de Valparaíso, Valparaíso 234000, Chile; 6 Departamento de Ciencias del Mar y Biología Aplicada, Universidad de Alicante, 03080 Alicante, Spain; 7 School of Biological and Marine Sciences, University of Plymouth, PL4 8AA Plymouth, UK; 8 Laboratory of Marine Biotechnology, Facultad de Química y Biología, Universidad de Santiago de Chile, Santiago 9170020, Chile

**Keywords:** mitogen-activated protein kinases, *Ulva compressa*, antioxidant, oxidative stress, metal chelator

## Abstract

Following the physiological complementary/parallel Celis-Plá et al., by inhibiting extracellular signal regulated kinases (ERK), c-Jun N-terminal kinases (JNK), and cytokinin specific binding protein (p38), we assessed the role of the mitogen-activated protein kinases (MAPK) pathway in detoxification responses mediated by chronic copper (10 µM) in *U. compressa*. Parameters were taken at 6, 24, and 48 h, and 6 days (d). H_2_O_2_ and lipid peroxidation under copper and inhibition of ERK, JNK, or p38 alone increased but recovered by the sixth day. By blocking two or more MAPKs under copper, H_2_O_2_ and lipid peroxidation decayed even below controls. Inhibition of more than one MAPK (at 6 d) caused a decrease in total glutathione (reduced glutathione (GSH) + oxidised glutathione (GSSG)) and ascorbate (reduced ascorbate (ASC) + dehydroascorbate (DHA)), although in the latter it did not occur when the whole MAPK was blocked. Catalase (*CAT*), superoxide dismutase (*SOD*), thioredoxin (*TRX*) ascorbate peroxidase (*APX*), dehydroascorbate reductase (*DHAR*), and glutathione synthase (*GS*), were downregulated when blocking more than one MAPK pathway. When one MAPK pathway was blocked under copper, a recovery and even enhancement of detoxification mechanisms was observed, likely due to crosstalk within the MAPKs and/or other signalling processes. In contrast, when more than one MAPK pathway were blocked under copper, impairment of detoxification defences occurred, demonstrating that MAPKs were key signalling mechanisms for detoxification in macroalgae.

## 1. Introduction

It has been recognised that besides metal exclusion/extrusion mechanisms, the main strategies by which macroalgae counteract metal excess in polluted environments are detoxification of bioavailable metals through the syntheses of chelating compounds and the inactivation of metal-mediated excess of reactive oxygen species (ROS) by the production and activities of antioxidants and antioxidant enzymes [1,2]. In green macroalgae (Ulvophyceae), metal chelation is importantly controlled by cysteine-rich compounds, among which the most important are the gene encoded metallothioneins and the enzymatically synthesised glutathione (GSH) and its poly-oligomers phytochelatins (PCs) [2,3]. GSH, in a recycling process with ascorbate (ASC), termed the Foyer–Halliwell–Asada cycle, is recognised as central to antioxidant metabolism in photoautotrophs, algae, and plants, and is one of the main mechanisms by which these organisms tolerate metal-mediated oxidative stress [2,4,5]. The process is controlled by complementary de novo synthesis and recycling of GSH and ASC. Briefly, GSH is synthesised by subsequent enzymatically mediated reactions controlled by γ-glutamate-cysteine ligase (γ-GCL) and glutathione synthase (GS), and in the case of ASC by l-galatose dehydrogenase (l-GDH) and l-galatonolactone dehydrogenase (l-GLDH) [4]. Moreover, the enzyme monodehydroascorbate reductase (MDHAR) uses GSH as a substrate to reduce dehydroascorbate (DHA) to ASC, also producing glutathione disulphide (GSSG). Then, the enzyme glutathione reductase (GR) reduces back GSSG to GSH using nicotinamide adenine dinucleotide phosphate (NADPH) [4]. Other enzymatic complexes also participate in the antioxidant metabolism, directly or indirectly linked with the GSH-ASC cycle. For instance, superoxide radicals (O_2_·^−^) are dismutated to hydrogen peroxide (H_2_O_2_), a lower oxidative-power ROS, by the enzyme superoxide dismutase (SOD) [6]. H_2_O_2_ is then reduced to water by either ascorbate peroxidase (APX) using ASC as electron donor, or by the enzyme catalase (CAT) [7]. Another important group of enzymes within the antioxidant metabolism of photoautotrophs is thioredoxins (TRXs), which principally reduce oxidised cysteine residues and split disulfide bonds [8].

Information on the role of antioxidant metabolism on copper-stressed green macroalgae is mostly derived from studies using the model *Ulva compressa*. For example, *U. compressa* from copper polluted sites, compared with non-impacted areas, of northern Chile displayed high levels of lipid peroxidation, enhanced activity of APX, increased ascorbate (as DHA), and consumption of glutathione pools [9]. Likewise, when exposed to chronic copper levels of 10 µM for up to 10 days (d) in the laboratory, *U. compressa* displayed increased levels of lipid peroxidation, accompanied by greater content of ASC, GSH, and PCs, higher expression of γ*-GCL*, *GS*, *L-GDH*, *L-GLDH*, *DHAR*, and *GR*, and increased transcripts and activities of the enzymes APX, GR, and SOD [10,11,12,13]. Similar induction of copper- and ROS-detoxification mechanisms have been reported in other *Ulva* species, including *U. lactuca* [14] and *U. fasciata* [15]. Together, these results indicate that the activation of antioxidant defences under chronic chronic copper excess is a transcriptionally regulated process in green macroalgae [2].

Although the main mechanisms by which green macroalgae withstand chronic chronic copper excess are now fairly well described, the cellular signalling pathways by which these organisms activate such metabolic machinery is poorly understood, and this is also true for exposure to environmental stress more generally. To date, the signalling pathways that have been observed to play a role in copper tolerance in green macroalgae relate to ROS, nitric oxide (NO), and calcium (Ca^+2^) signalling. The potential participation of the mitogen-activated protein kinases (MAPKs) has been proposed, but not fully confirmed [2,12]. MAPKs are serine/threonine kinases following a cascade of activations from MAPK kinase kinase (MAPKKK), MAPK kinase (MAPKK) to MAPK, thus finally inducing gene expression [1,16,17]. There are three main sub-pathways within the MAPKs, these ending in the MAPK ERK (extracellular signal regulated kinases), JNK (c-Jun *N*-terminal kinases), and p38 (cytokinin specific binding protein) [1,16,17]. While a full description of the mechanistic contribution of these MAPK pathways to tolerance machinery under environmental stress in photoautotrophs has not been elucidated, their presence and induction have been observed in both micro and macroalgae [18,19,20,21].

Here, we report on a holistic investigation to disclose for the first time in photoautotrophs the role of different pathways within the MAPKs to withstand environmental stress, in this case caused by chronic copper excess in green macroalgae. The green seaweed *U. compressa* under 10 µM of copper was exposed to specific inhibitors of MAPK pathways, ERK, JNK, and/or p38, with measurements taken at different time points throughout the experiment (6 h, 24 h, 48 h, and 6 d). In a complementary investigation, we demonstrated that under chronic copper excess when two or more MAPK pathways were inhibited, there was a general trend of decreasing intracellular copper concentrations when compared to exposure only to copper [1]. Moreover, the lowest levels of photosynthetic performance, as determined from measurements of photosynthetic efficiency (α_ETR_), productivity (ETR_max_), and saturation of the irradiance (Ek_ETR_), were under copper in the presence of two or more MAPK inhibitors, [1]. Coincidentally, on the sixth d non-photochemical quenching (NPQ_max_) increased in treatments with copper and a mixture of MAPK inhibitors, confirming that the perturbed electron transport in PSII was being countered by the dissipation of heat [1]. These data suggest that by blocking most of the MAPK pathways, an alternative cellular signalling pathway(s) may be over-stimulated, thus enhancing mechanisms of copper exclusion/extrusion. Moreover, results indicate that the MAPK pathways may have a central role in photosynthetic performance, even under the presence of environmental perturbation (in this case, chronic copper excess) [1]. Here, we extend this study to investigate the participation of MAPK pathways in the reactive oxygen metabolism (ROM) of *U. compressa* under chronic copper excess. More specifically, we evaluated parameters of oxidative stress and damage, concentrations of glutathione (GSH and GSSG), and ascorbate (ASC and DHA), as well as the expression of enzymes involved in antioxidant metabolism (*APX*, *CAT*, *SOD*, *GS*, *DHAR*, and *TRX*).

## 2. Results

### 2.1. Hydrogen Peroxide Levels

**Figure 1:**
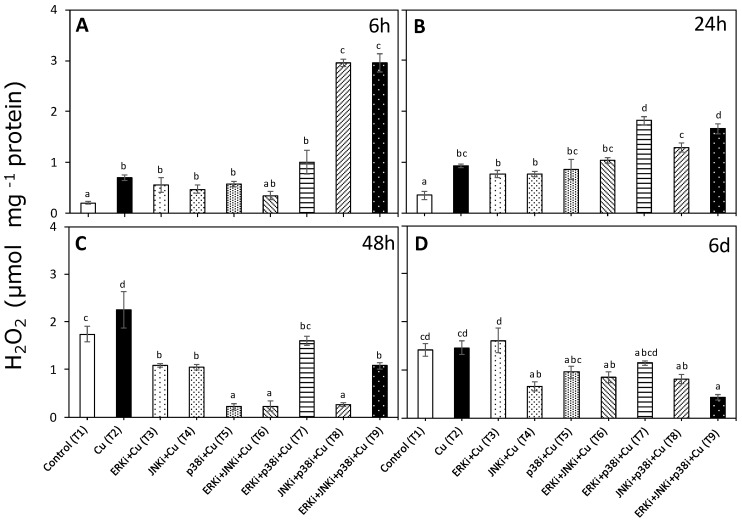
Hydroxide peroxide (H_2_O_2_) levels in *U. compressa* under copper and/or exposure to mitogen-activated protein kinases (MAPK) inhibitors. Treatments consisted in: T1) control only with seawater; T2) solely copper exposure as 10 µM of CuSO_4_ (Cu); T3) copper + 5 µM MAPK extracellular signal regulated kinases (ERK) inhibitor PD98059 (Cu + ERKi); T4) copper + 5 µM MAPK c-Jun *N*-terminal kinases (JNK) inhibitor SP600125 (Cu + JNKi); T5) copper + MAPK cytokinin specific binding protein (p38) inhibitor SB203580 (Cu + p38i); T6) Cu + ERKi + JNKi; T7) Cu + ERKi + p38i; T8) Cu + p38i + JNKi; and T9) Cu + ERKi + JNKi + p38i. Samples were analysed after 6 h (**A**), 24 h (**B**), 48 h (**C**), and 6 d (**D**) treatments. Different letters represent significant difference at 95% confidence interval (*p* < 0.05). Plots are represented as mean ± SE (*n* = 3).

The levels of H_2_O_2_ were measured as an indicator of ROS imbalance (Figure 1). At 6 h, all treatments compared with control samples induced a significantly higher accumulation of H_2_O_2_, especially treatments with combinations of inhibitors that included p38i (Figure 1A). Treatments that had significantly higher levels than those of T2 (copper only) were: Cu + ERKi + JNKi (T6) and Cu + ERKi + JNKi + p38i (T9). Similar patterns were observed at 24 h, although the Cu + ERKi + p38i (T7) and Cu + ERKi + JNKi + p38i (T9) treatments were the ones with significantly higher levels of H_2_O_2_ (Figure 1B). Inverted patterns could be detected at 48 h and 6 d, where concentrations of H_2_O_2_ in all treatments containing copper and inhibitors were either the same as or significantly lower than control and copper-only treatments (Figure 1C,D).

### 2.2. Lipid Peroxidation

**Figure 2:**
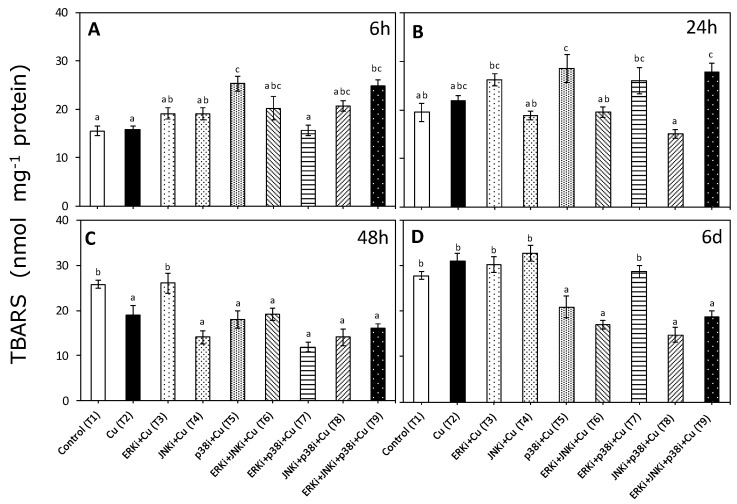
Thiobarbituric acid reactive substances (TBARS) accumulation in *U. compressa* under copper and/or exposure to MAPK inhibitors. Treatments consisted in: T1) control only with seawater; T2) solely copper exposure as 10 µM of CuSO_4_ (Cu); T3) copper + 5 µM MAPK ERK inhibitor PD98059 (Cu + ERKi); T4) copper + 5 µM MAPK JNK inhibitor SP600125 (Cu + JNKi); T5) copper + MAPK p38 inhibitor SB203580 (Cu + p38i); T6) Cu + ERKi + JNKi; T7) Cu + ERKi + p38i; T8) Cu + p38i + JNKi; and T9) Cu + ERKi + JNKi + p38i. Samples were analyzed after 6 h (**A**), 24 h (**B**), 48 h (**C**) and 6 d (**D**) treatments. Different letters represent significant difference at 95% confidence interval (*p* < 0.05). Plots are represented as mean ± SE (*n* = 3).

Exposure to copper did not induce significantly higher levels of TBARS than controls at 6 and 24 h, nor did the majority of the treatments containing inhibitors, however copper treatments with p38 (T5) and all three inhibitors (T9) had significantly higher levels of TBARS (Figure 2A,B). At 48 h, in treatments with copper without and with inhibitors (except with ERKi alone), there were significant decreases in TBARS compared to the controls, although there were no significant differences between these treatments (Figure 2C). At day 6, no differences could be detected between the controls and treatments with only copper and those containing copper and ERKi (T3), JNKi (T4), and ERKi + p38i (T7). In all other treatments with copper plus inhibitors, there were significantly lower levels of TBARS than controls and under copper only, but with no differences between them (Figure 2D).

### 2.3. Levels of Reduced and Oxidised Glutathione

**Figure 3:**
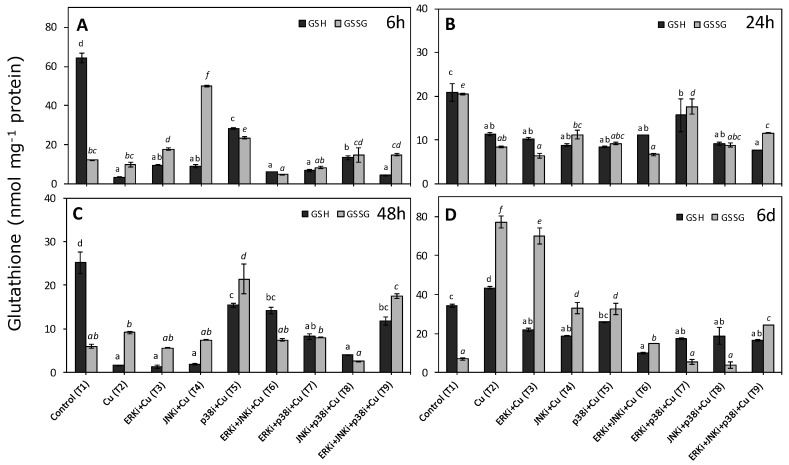
Total reduced (GSH) and oxidised glutathione (GSSG) content in *U. compressa* under copper and/or exposure to MAPK inhibitors. Treatments consisted in: T1) control only with seawater; T2) Solely copper exposure as 10 µM CuSO_4_ (Cu); T3) copper + 5 µM MAPK ERK inhibitor PD98059 (Cu + ERKi); T4) copper + 5 µM MAPK JNK inhibitor SP600125 (Cu + JNKi); T5) copper + MAPK p38 inhibitor SB203580 (Cu + p38i); T6) Cu + ERKi + JNKi; T7) Cu + ERKi + p38i; T8) Cu + p38i + JNKi; and T9) Cu + ERKi + JNKi + p38i. Samples were analysed after 6 h (**A**), 24 h (**B**), 48 h (**C**), and 6 d (**D**) treatments. Different letters represent significant difference at 95% confidence interval (*p* < 0.05). Plots are represented as mean ± SE (*n* = 3).

At 6 h, there was a significant decrease of total glutathione and GSH in all treatments compared to controls (Figure 3A). Moreover, in most treatments with copper (T2) and copper plus inhibitors (T3-T9) there were the same or significantly lower levels of GSSG than controls; the exceptions were copper with single MAPK inhibitor treatments (T3, T4 and T5) in which significantly higher GSSG concentrations were measured (Figure 3A). It is important to highlight that controls presented over 4-fold levels of GSH in comparison to GSSG, whereas the majority of the other treatments had higher GSSG than GSH (Figure 3A). At 24 h, similar concentrations within each treatment were recorded for GSH and GSSG; in addition, all treatments containing only copper (T2) and copper with inhibitors (T3-T9) displayed significantly lower levels of GSH and GSSG than controls (Figure 3B). Only in the case of treatment Cu + ERKi + p38i (T7), were concentrations of GSSG higher than in copper alone (T2) (Figure 3B). As at 6 h, at 48 h the levels of GSH were 4 times higher than GSSG in controls (Figure 3C). In treatments with only copper, copper with either ERKi or JNKi, and copper with all the blocked MAPKs, GSSG concentrations were higher than those of GSH (Figure 3C). In most treatments containing copper, concentrations of GSH were significantly lower than controls; the exception was treatment under copper and either ERKi (T3) or JNKi (T4) (Figure 3C). There were no clear patterns in GSSG across treatments, although in treatments with copper and either p38 or all three inhibitors, concentrations of GSSG were significantly higher than controls and copper only treatments (Figure 3C). By the 6th d of the experiment, GSH concentrations in the copper only treatment were significantly higher than controls, and levels in the controls were significantly higher than when inhibitors were present; there was no clear pattern amongst these latter treatments (Figure 3D). Similarly for significantly higher concentrations than in controls were measured in the copper only and copper plus ERKi, both having the largest pool of total glutathione (Figure 3D).

### 2.4. Concentrations of Reduced and Oxidised Ascorbate

**Figure 4:**
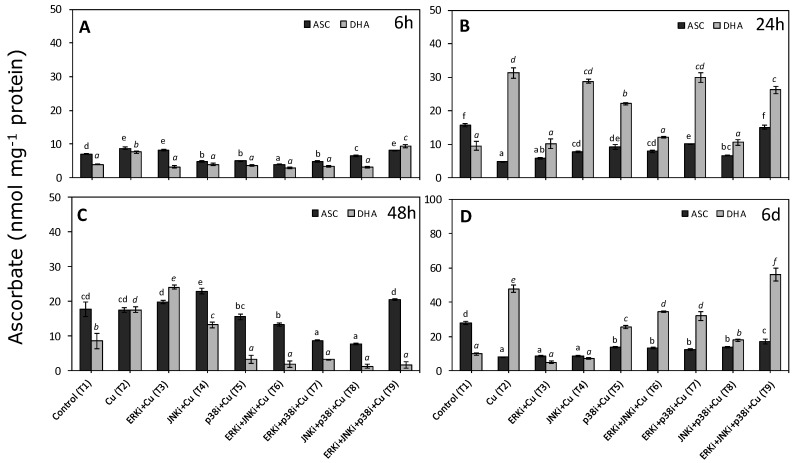
Total reduced ascorbate (ASC) and dehydroascorbate (DHA) concentrations in *U. compressa* under copper and/or exposure to MAPK inhibitors. Treatments consisted in: T1) control only with seawater; T2) solely copper exposure as 10 µM of CuSO_4_ (Cu); T3) copper + 5 µM MAPK ERK inhibitor PD98059 (Cu + ERKi); T4) copper + 5 µM MAPK JNK inhibitor SP600125 (Cu + JNKi); T5) copper + MAPK p38 inhibitor SB203580 (Cu + p38i); T6) Cu + ERKi + JNKi; T7) Cu + ERKi + p38i; T8) Cu + p38i + JNKi; and T9) Cu + ERKi + JNKi + p38i. Samples were analysed after 6 h (**A**), 24 h (**B**), 48 h (**C**), and 6 d (**D**) treatments. Different letters represent significant difference at 95% confidence interval (*p* < 0.05). Plots are represented as mean ± SE (*n* = 3).

The lowest concentrations of all ASC, DHA, and total ascorbate pool were measured at 6 h (Figure 4A). ASC levels were higher than in controls in treatments with only copper, Cu + ERKi (T3), and Cu + ERKi + JNKi + p38i (T9) (Figure 4A). The remainder of the treatments always had significantly lower levels of ASC compared to the controls (Figure 4A). There was a similar trend for DHA, although levels were only higher than in controls for treatment-only copper (T2) and copper with all combined inhibitors (T9) (Figure 4A). With the exception of T9, higher ASC concentrations were significantly higher than those of DHA (Figure 4A). At 24 h, the total ascorbate pool, mainly due to DHA, increased considerably compared to 6 h in all treatments containing only copper and copper with inhibitors (Figure 4B). Excluding the treatment with copper and all inhibitors combined (T9), ASC levels were always significantly lower in treatments containing copper than the controls (Figure 4B). In contrast, treatments containing copper had higher DHA concentrations than the controls, the exceptions being those with ERKi (T3, T6, T7, and T9) (Figure 4B). At 48 h, the general trend was for higher ASC than DHA levels, except for treatments with only copper and with copper and ERKi (Figure 4C). Treatments combining copper with two inhibited MAPK pathways had significant lower levels of ASC than the controls, whereas in treatments with only copper, single inhibitor exposure, ERKi (T3) or p38i (T5), and all three inhibitors (T9) there were no significant differences with controls, however in only Cu + JNKi (T4) was ASC significantly higher than the controls (Figure 4C). Concentrations of DHA were significantly higher under exposure to copper alone (T2), copper and ERKi (T3), and copper and JNKi (T4), compared to the controls (T1) (Figure 4C). The lowest levels of DHA were measured in all treatments having combinations of inhibitors (T6-T9) and in Cu + p38i (T5), all being significantly lower than controls (Figure 4C). The highest levels of total ascorbate were measured on d 6, with concentrations of 60 and 70 nmol mg^−1^ protein in treatments with only copper (T2) and copper plus all three inhibitors (T9), respectively (Figure 4D). Treatments containing copper generally had higher DHA than ASC levels, with the exceptions of copper and ERKi (T3) or JNKi (T4) (Figure 4D). Finally, in ascending order, the highest concentrations of DHA were observed at solely copper and subject to copper combined with all inhibitors (Figure 4D).

### 2.5. Gene Expression Profiles

**Figure 5:**
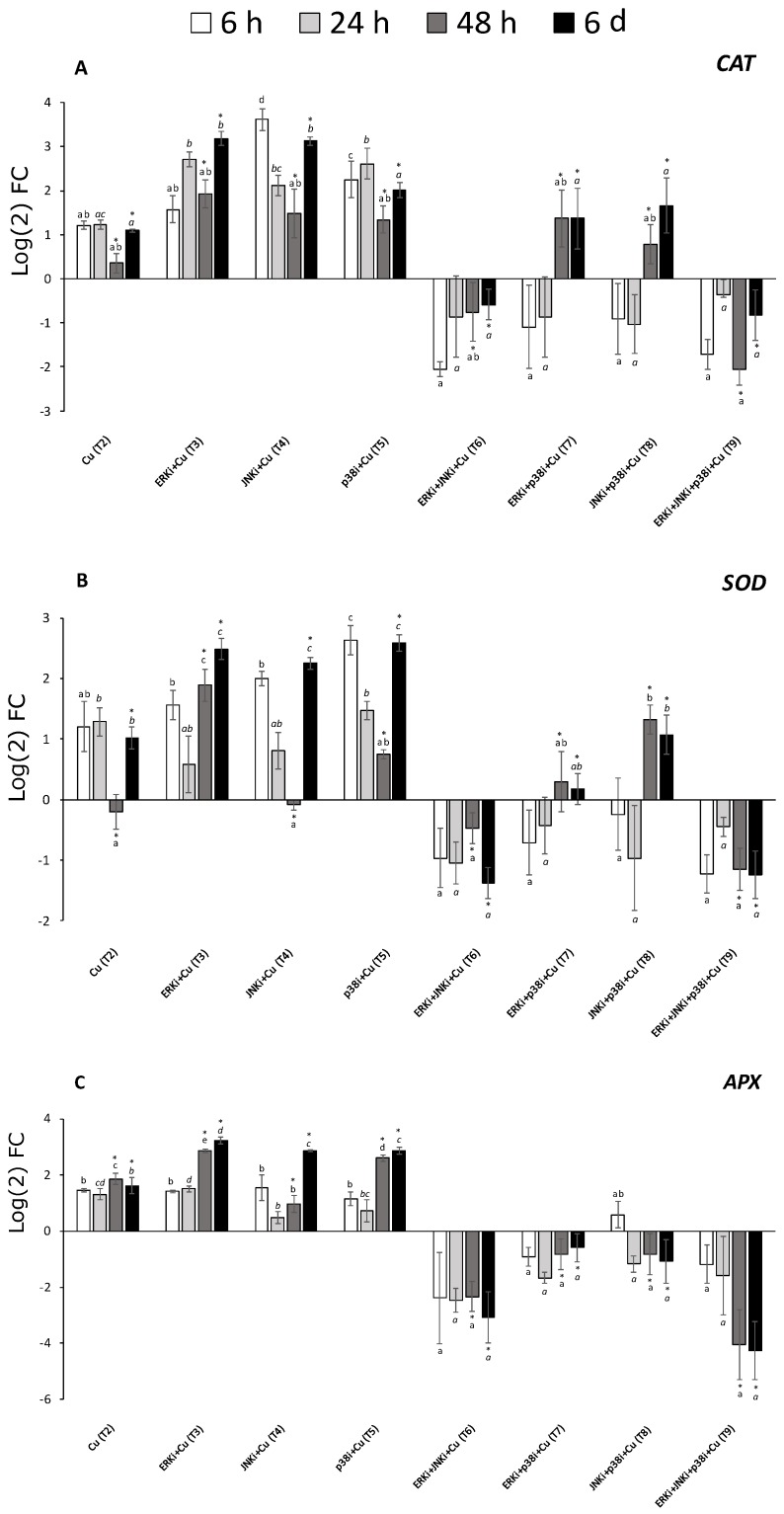
Relative expression of the genes catalase (*CAT*; **A**), superoxide dismutase (*SOD*; **B**), dehydroascorbate reductase (*DHAR*; **C**) in *U. compressa* under copper and/or exposure to MAPK inhibitors. Expression was relative to the levels of transcripts in *U. compressa* under control conditions. Treatments consisted in: T1) solely copper exposure as 10 µM of CuSO_4_ (Cu); T2) copper + 5 µM MAPK ERK inhibitor PD98059 (Cu + ERKi); T3) copper + 5 µM MAPK JNK inhibitor SP600125 (Cu + JNKi); T4) copper + MAPK p38 inhibitor SB203580 (Cu + p38i); T5) Cu + ERKi + JNKi; T6) Cu + ERKi + p38i; T7) Cu + p38i + JNKi; and T8) Cu + ERKi + JNKi + p38i. Samples were analysed after 6 h (A), 24 h (B), 48 h (C), and 6 d (D) treatments. Different letters, in italics and/or with asterisk represent significant difference at 95% confidence interval (*p* < 0.05) within each experimental time. Plots are represented as mean ± SE (*n* = 3).

**Figure 6:**
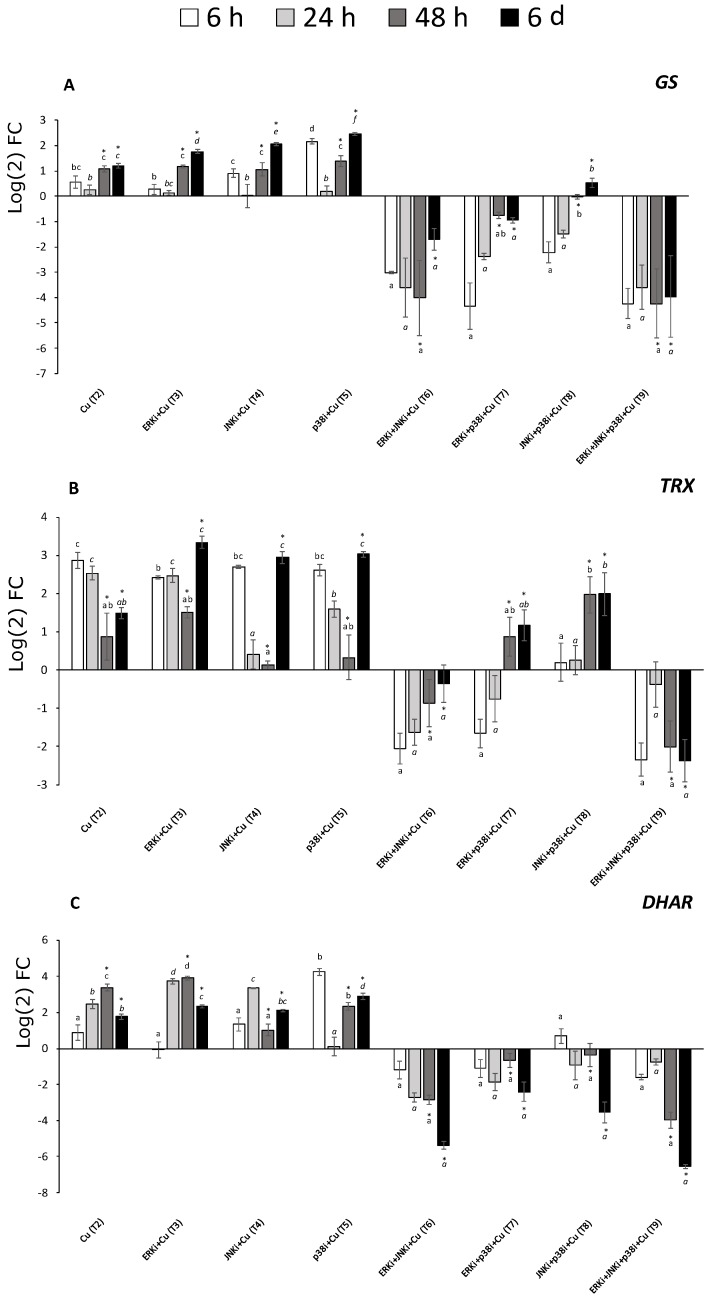
Relative expression of the genes glutathione synthase (*GS*; **A**), thioredoxin (*TRX*; **B**), and dehydroascorbate reductase (*DHAR*; **C**) in *U. compressa* under copper and/or exposure to MAPK inhibitors. Expression was relative to the levels of transcripts in *U. compressa* under control conditions. Treatments consisted in: T1) solely copper exposure as 10 µM of CuSO_4_ (Cu); T2) copper + 5 µM MAPK ERK inhibitor PD98059 (Cu + ERKi); T3) copper + 5 µM MAPK JNK inhibitor SP600125 (Cu + JNKi); T4) copper + MAPK p38 inhibitor SB203580 (Cu + p38i); T5) Cu + ERKi + JNKi; T6) Cu + ERKi + p38i; T7) Cu + p38i + JNKi; and T8) Cu + ERKi + JNKi + p38i. Samples were analysed after 6 h (A), 24 h (B), 48 h (C), and 6 d (D) treatments. Different letters, in italics and/or with asterisk represent significant difference at 95% confidence interval (*p* < 0.05) within each experimental time. Plots are represented as mean ± SE (*n* = 3).

Similar behaviour could be described in the expression of the studied genes. Under copper alone (T2), all genes, at most time points, were upregulated. When the alga was subjected to copper and single inhibitor exposure (T3-T5), the general trend was also upregulation at most time points with an oscillatory pattern of decrease at 24 and 48 h and then increase at 6 d, although there were certain exceptions to this, as for *CAT* and *DHAR* (Figure 5A,C, respectively). Conversely, when two or more MAPK pathways were blocked, the general trend was downregulation, even though at most time periods the differences were not significant (Figure 5 and Figure 6). There were several exceptions to this general trend for example, at 48 h and 6 d *CAT, SOD*, and *TRX* were upregulated in treatments containing copper and p38i with either ERKi (T7) or JNKi (T8) (Figure 5A,B, and Figure 6A, respectively).

### 2.6. Principal Coordinate Ordination (PCO) Analysis

**Figure 7:**
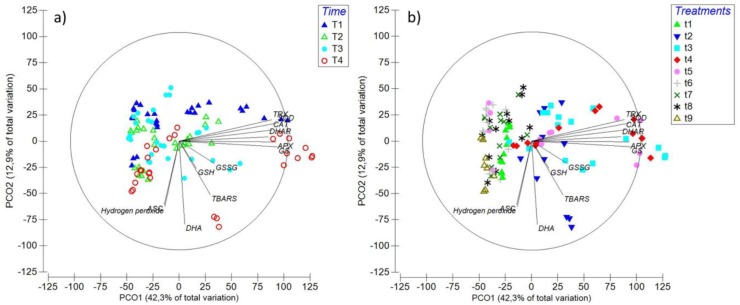
Principal components ordination (PCO) analysis diagrams in relation with time (**a**): T1: 6 h, T2: 24 h, T3: 48 h, and T4: 6 d; and treatments (**b**): t1: Control only with seawater, t2: Solely copper exposure as 10 µM of CuSO_4_ (Cu), t3: copper + 5 µM MAPK ERK inhibitor PD98059 (Cu + ERKi), t4: Copper + 5 µM MAPK JNK inhibitor SP600125 (Cu + JNKi), t5: Copper + MAPK p38 inhibitor SB203580 (Cu + p38i), t6: Cu + ERKi + JNKi, t7: Cu + ERKi + p38i, t8: Cu + p38i + JNKi, and t9: Cu + ERKi + JNKi + p38i. Vectors overlay (Sperman rank correlation) indicate the relationship between the PCO axes and the parameters H_2_O_2_, TBARS (thiobarbituric acid reactive substance), GSH (reduced glutathione), GSSG (oxidised glutathione), ASC (reduced ascorbate), DHA (dehydroascorbate), and relative gene expression catalase (*CAT*), superoxide dismutase (*SOD*), dehydroascorbate reductase (*DHAR*), thioredoxin (*TRX*), ascorbate peroxidase (*APX*), and glutathione synthase (*GS*).

Considering all whole data presented in this article, evidence of clustering of the measured parameters in relation to time period and treatment can be discerned from the PCO (Figure 7). In terms of time, there was an association after the 6th d in the expression of all studied genes, in agreement with the general pattern of increase in transcripts towards the end of the experiment (Figure 7a). Another grouping between GSH, GSSG, DHA, and thiobarbituric acid reactive substances (TBARS) is evident, albeit with no clear trend between time periods, that is attributable to the pattern of decrease in treatments containing copper with two or more MAPK inhibitors (T6-T9) (Figure 7a). The final cluster is between ASC and H_2_O_2_, which can be ascribed to the oscillatory pattern of increase and decrease in treatments containing copper and mixed inhibitors (T6-T9) between time periods (Figure 7a). Furthermore, certain clusters can be detected within different treatments, particularly in those with copper and two or more MAPK inhibitors (T6-T9), which is in agreement, at least in part, with the marked general patterns in gene expression, and the parameters of TBARS, glutathione, and ascorbate (Figure 7b).

**Figure 8:**
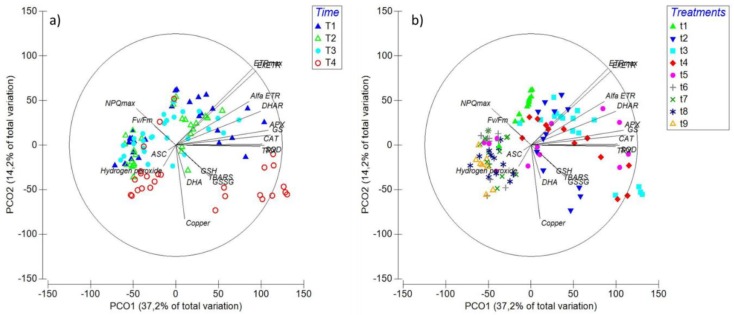
Principal Components Ordination (PCO) analysis diagrams considering the data covered in this article and in the parallel/complementary Celis-Plá et al. [1]. PCOs are related with time (**a**): T1: 6 h, T2: 24 h, T3: 48 h, and T4: 6 d; and treatments (**b**): t1: control only with seawater, t2: solely copper exposure as 10 µM CuSO_4_ (Cu), t3: copper + 5 µM MAPK ERK inhibitor PD98059 (Cu + ERKi), t4: copper + 5 µM MAPK JNK inhibitor SP600125 (Cu + JNKi), T5: copper + MAPK p38 inhibitor SB203580 (Cu + p38i), t6: Cu + ERKi + JNKi, t7: Cu + ERKi + p38i, t8: Cu + p38i + JNKi, and t9: Cu + ERKi + JNKi + p38i. Vectors overlay (Sperman rank correlation) indicate the relationship between the PCO axes and the parameters H_2_O_2_, TBARS (thiobarbituric acid reactive substance), GSH (reduced glutathione), GSSG (oxidised glutathione), ASC (reduced ascorbate), DHA (dehydroascorbate), and relative gene expression catalase (*CAT*), superoxide dismutase (*SOD*), dehydroascorbate reductase (*DHAR*), thioredoxin (*TRX*), ascorbate peroxidase (*APX*), and glutathione synthase (*GS*). Furthermore, the physiological variables intracellular copper accumulation (copper photoinhibition (*Fv*/*Fm*) productivity (ETRmax), efficiency (αETR) and saturation of irradiance (EkETR), accompanied by higher non-photochemical quenching (NPQmax) [1].

Taking into account the results presented in this article and those of the parallel-complementary paper Celis-Plá et al. [1], a second PCO was prepared combining the whole dataset from of both articles (Figure 8). Clear trends are apparent in the grouping between gene expression and photosynthetic parameters associated with electron transport rates (ETR_max_, α_ETR_ and EkETR), which have in common the marked decrease under copper and mixed inhibitor treatments (T6-T9) (Figure 8). The GSH, GSSH, DHA and TBARS cluster was maintained but with the addition of intracellular copper accumulation [1]; this obey to, not as marked as in gene expression and ETR associated parameters, a decrease in concentrations upon exposure to copper and mixed MAPK inhibitors (T6-T9) (Figure 8). The grouping between ASC and H_2_O_2_ is also maintained but with the addition of the photosynthetic parameters of photoinhibition, Fv/Fm and photoprotection (NPQ_max_); the latter was the most isolated as at 6 d the trend was a general increase in copper treatments containing combined inhibitors (T6-T9) compared to that with solely copper (T2) (Figure 8). Upon time, clustering was mostly observed at d 6, attributed to the numeric dominance of ETR related parameters, levels of transcripts, GSH, GSSG, DHA and TBARS (Figure 8a). Grouping was repeated mostly within treatments considering copper and two or more combined MAPK inhibitors (T6-T9) (Figure 8b).

## 3. Discussion

In this investigation, we exposed the green macroalga *Ulva compressa* to chronic copper excess (10 µM), and the relevance of the three main MAPK pathways, those ending in ERK, JNK, and p38, were studied upon their role within detoxification mechanisms associated with metal chelation, but mostly, with antioxidant responses. We observed that up to 24 h, levels of ROS H_2_O_2_ increased markedly compared to the controls under the treatment with only copper, and that in treatments blocking p38 under single or combined exposure (T5, T7-T9) with other inhibitors concentrations were the same or even higher than under copper alone (T2). That pattern at 48 h was inversed, and by the sixth day of the experiment, treatments with copper and solely p38i (T5) and combined inhibitors, excepting ERKi + p38i (T7), displayed even lower levels of H_2_O_2_ than under copper-only treatments and controls. Interestingly, almost the same pattern was observed for lipid peroxidation. For antioxidants, most treatments with copper displayed higher levels of GSSG and DHA than their reduced forms GSH and ASC, respectively, although this was more evident for ascorbate. Towards the end of the experiment, while the greatest concentrations of total glutathione were observed with copper alone (T2), the lowest levels were measured under copper and mixed MAPK inhibitor treatments (T6-T9). In contrast, the ascorbate pool was highest in the treatment with copper and all three MAPK pathways blocked, followed by copper alone (mostly due to DHA). Clear general patterns could be observed among the expression of the genes *CAT*, *SOD*, *DHAR*, *TRX*, *APX*, and *GS*. Upregulation, sometimes higher than under copper alone, was detected in treatments with copper and ERKi (T3), JNKi (T4), or p38i (T5), and mostly downregulation when the alga was subjected to copper and more than one inhibited MAPK pathway (T6-T9).

It is well recognised that chronic copper excess can induce oxidative stress and, in severe cases, cause damage in photoautotrophs [2,4]. Although chronic copper excess can produce oxidative stress in *U. compressa* under field conditions [9,22], this has also been described for other green macroalgae species subject to laboratory-controlled experiments. For example, it has been detected that *Ulva fasciata* exposed to copper concentrations of over 20 µM for 4 d displayed increased lipid peroxidation [15]. Notwithstanding the latter result, in this investigation no differences in lipid peroxidation were detected between controls and exposure to copper alone over the experimental period. While at most time periods levels of H_2_O_2_ were higher under copper alone compared to controls, the data suggest that these concentrations of H_2_O_2_ by themselves, or in combination with other ROS, were insufficient to cause oxidative damage, and ROS concentrations remained within homeostatic-tolerable levels. A different pattern was observed when blocking certain MAPK pathways, which started at 48 h but became more marked by 6 d. Inhibiting p38 (T5), ERK + JNK (T6), JNK + p38 (T8), and ERK + JNK + p38 (T9) under chronic copper excess, coincidentally decreased both H_2_O_2_ and lipid peroxidation relative to controls (T1) and under copper-only treatment (T2). The information demonstrates that under short-term copper exposure (especially at 6 h), the inhibition of more than one MAPK pathway causes increased oxidative stress and damage. However, over longer periods of copper exposure (mainly at 6 d), the patterns observed during short-term exposure become generally inverted, with resilient (adaptation response) lower levels of oxidative stress and damage in certain single and combined MAPK inhibitor treatments.

To counteract oxidative stress, the main mechanism recognised in photoautotrophs is associated with the glutathione-ascorbate, or Foyer-Halliwell-Asada, pathway [4,23]. As in previous investigations [13], *U. compressa* displayed a higher total glutathione pool under chronic copper excess in relation to controls, although at late exposures (6 d). At 48 h and beyond, patterns of higher GSSG upon GSH were observed in copper-exposed macroalgae, which is coincident with an induction of ROS over-production and GSH oxidation, indeed, a decrease in GSH/GSSG ratios have been recorded in several photoautotrophs under chronic copper excess and is fairly recognised as a good indicator of an oxidative stress condition [2,4,5,23,24]. In the presence of copper and MAPK inhibitors, most consistent trends were observed at 6 d. In most treatments with copper and one or more MAPK blocked pathways, with the exceptions of ERKi + p38i (T7) and JNKi + p38i (T8), GSH levels were always lower than GSSG. In spite of the latter, the total glutathione pool was always lower in treatments containing MAPK inhibitors (T3–T9) compared to that with solely copper (T2), and especially in those inhibiting more than one MAPK pathway (T6–T9). Similar tendencies were detected for ascorbate, particularly after 6 d. In most of the copper exposure treatments, ASC levels were higher than DHA, with the exception of treatments blocking ERK (T3) or JNK (T4). Total ascorbate was generally lower under copper and suppression of MAPK pathways, drawing certain exceptions (see results). Chronic copper excess also induced upregulation of *CAT*, *SOD*, *DHAR*, *TRX*, *APX*, and *GS* at most time periods and generally increasing upon exposure time. A similar behaviour in the expression of these genes was detected when chronic copper excess was applied and ERK (T3), JNK (T4), or p38 (T5) were blocked, with a recovery process throughout the experimental period (more details in results). In contrast, when more than one MAPK pathway was inhibited (T6-T9), at most of the experimental times the studied genes displayed downregulation. An interesting feature of our investigation lies in the fact that both GS expression and GSH levels in *U. compressa* displayed similar behaviour among treatments and time. Previous investigations under high copper in *U. compressa* have shown that the increased induction of GS and GSH is accompanied by a parallel increment in the synthesis and polymerisation of GSH oligomers PCs [13]. Thus, this information suggests that MAPKs may also play an important role in the production of metal-chelating thiols, not only GSH but also PCs. Although records about the regulation of the glutathione-ascorbate cycle through MAPK signalling are scarce in eukaryotes, and especially in photoautotrophs, Karuppanapandian and Kim [25] observed that the plant *Brassica juncea* under 100 µM of cobalt, activated 44 kDa and 46 kDa MAPKs, which was accompanied by an increases in GSH, GSSG, ASC, and DHA. Furthermore, Shan and Sun [26] by blocking ERK (with PD98059 and U0126), observed that jasmonic acid-mediated production of nitric oxide activated ERK which, in turn, induced increased activities of APX, GR, DHAR, MDHAR, γ-GCL, and L-GLDH, which also enhanced syntheses of ASC and GSH. Moreover, Qi et al. [27] detected that by inhibiting ERK (PD98059) in the plant *Cucumis sativus* under nitrate excess induced decreased activities of SOD, CAT, APX, and peroxidases (POD), being responsible, at least in part, for the observed greater lipid peroxidation and H_2_O_2_ content. To the extent of our knowledge, there are no published studies describing the role of the three main MAPK pathways, ERK, JNK, and p38 alone or complemented in the antioxidant metabolism of photoautotrophs under environmental stress. In our investigation, we demonstrated that the whole MAPK pathway has an important role in the antioxidant metabolism of macroalgae. The data shows that under single and combined inhibitors of the three main MAPK pathways, there is a progressive affection of glutathione production. Although all three MAPK pathways show to have an influence in GSH production, JNK and p38 appear to be more relevant than ERK. Furthermore, when more than one MAPK pathway is affected, total glutathione is almost impaired. Similar effects can be observed for ascorbate, however, this was observed at late copper exposure, and the main pathways regarding its production were related to the ERK, JNK, and JNK + p38 MAPK pathways. In relation to the expression of genes involved in antioxidant metabolism and the synthesis of metal chelators, when one of the three pathways is affected independently, in time it appears to enhance the upregulation mediated by chronic copper excess. Crosstalk is a common phenomenon among signalling mechanisms and has been observed within MAPK pathways as well as with other signalling processes as those related to phytohormones, calcium, NO, ROS, and among others [28,29,30,31]. In this context, it is possible that to counteract the inability of certain MAPK pathways, others MAPKs and/or more cellular signalling mechanisms could be triggered and elicit an intensified transcriptomic response in *U. compressa*. Although no similar investigations have been conducted in photoautotrophs, ERK inhibition in human melanoma cells have demonstrated to be counteracted through the activation of alternative signalling pathways, such as those mediated by phosphoinositide 3-kinases (PI3K) and Protein kinase B (Akt) [32,33,34,35,36]. Furthermore, inhibition of ERK induces an upregulation of the transcription factor Forkhead box D3 (*FOXD3*), involved in the development of embryo stem cells, mediated by the activation of PI3K and receptor tyrosine-protein kinase (ErbB3) [37,38]. Considering that in our investigation there was mostly gene downregulation when more than one MAPK pathway was blocked under chronic copper excess, the information suggests that the gene over-expression in *U. compressa* is promoted mainly by a crosstalk within the whole MAPK signalling pathway. On the other hand, it could be hypothesised that gene repression under copper and combined MAPK inhibitors is caused by the low levels of intracellular copper detected in these treatments in Celis-Plá et al. [1]. Indeed, it has been observed that the expression of the enzymes SOD, CAT, and APX had a positive correlation with levels of intracellular copper in the brown macroalga *Ectocarpus siliculosus* [5,39]. However, this explanation appears unlikely considering that the decline of antioxidant defences in treatments with copper and combined MAPK inhibitors most of the time reached levels even below control conditions. Thus, MAPK signalling demonstrates to be an important mechanism for the activation of copper-detoxification defences in *U. compressa*.

Multivariate analyses confirmed that the data contained in this article was importantly influenced by time, but mostly by treatments. Furthermore, considering the results of the complementary/parallel Celis-Plá et al. [1], the important agreement between physiological, biochemical, and transcriptomic responses in MAPK-intervened *U. compressa* can be highlighted. In this regard, general tendencies demonstrate that the inactivation of single MAPK pathways ending in ERK, JNK, and p38 subject to high copper can be progressively counteracted by complementary biological strategies, which appear to be related to crosstalk within the MAPKs and/or with other signalling pathways. Despite the latter, when more than one of these MAPK pathways is disabled under chronic copper excess, biological impairment start to manifest, which is traduced in diminished photosynthetic activity [1], ROS-overproduction, increased oxidative damage, and affected production and regulation of antioxidant mechanisms. These investigations provide the first complete evidence on the importance of the MAPK pathway to counteract environmental stress in photoautotrophs, and further confirm that copper-detoxification mechanisms are importantly founded on transcriptionally regulated metabolic and physiological processes in green macroalgae. Certainly, additional research on the whole transcriptome of *U. compressa* subject to modified MAPK activation/inactivation will ascertain on open questions regarding green macroalgae biological strategies to withstand stress, in this case, induced by chronic copper excess.

## 4. Methodology

### 4.1. Samples Collection and Culture Conditions

*Ulva compressa* (Chlorophyta, Ulvaceae) was sampled from Cachagua beach, Valparaíso Region, Chile (32°34’59 S; 71°26’16 W). Algae material was washed with 2 µm of filtered seawater and stored in plastic containers with seawater under constant aeration at 16 °C. The photoperiod cycle was 12:12 (day/night) with daylight conditions of 120 µmol m^2^·s^−1^ of photosynthetic active radiation (PAR). Algae were acclimated for 48 h before experiments.

### 4.2. Copper and MAPK Inhibitors Treatments

Following the acclimation period, 5 g of fresh weight (FW) *U. compressa* were separated in different plastic containers with 200 mL of filtered (2 µm) and autoclaved seawater. Nine different treatments, with three replicates each, were conducted as: T1) control with just seawater; T2) only copper exposure as 10 µM of CuSO4 (Sigma-Aldrich, St. Louis, MO, USA) (Cu); T3) copper + 5 µM MAPK ERK inhibitor PD98059 (Tocris Bioscience, St. Louis, MO, USA) (Cu + ERKi); T4) copper + 5 µM MAPK JNK inhibitor SP600125 (Tocris Bioscience, St. Louis, MO, USA) (Cu + JNKi); T5) copper + MAPK p38 inhibitor SB203580 (Tocris Bioscience) (Cu + p38i); T6) Cu + ERKi + JNKi; T7) Cu + ERKi + p38i; T8) Cu + p38i + JNKi; and T9) Cu + ERKi + JNKi + p38i. All treatments were performed under the same environmental conditions mentioned in Section 2.1. Negative controls to assess the effectiveness of the ERK, JNK, and p38 inhibitors were conducted by measuring the expression of *SOD* and *CAT* (protocols in Section 2.5 and Section 2.6) in the absence of copper (Figure S1) and these were complemented by negative controls contained as Supplementary Materials in Celis-Pla et al. [1]. Even though the inhibitors used were first developed to target human MAPKs, this and prior investigations demonstrate their successful application to other eukaryote models given the conserved nature of MAPKs (further details) in [1].

During the experiments, sub-samples were taken from each treatment at 6 h, 24 h, 48 h, and 6 d, immediately frozen in liquid nitrogen and stored at −80 °C for biochemical and molecular analyses.

### 4.3. Hydrogen Peroxide (H_2_O_2_) Quantification

Concentrations of H_2_O_2_ were quantified using a modification of the colorimetric method by which H_2_O_2_ is induced by its interaction with potassium iodide (KI), as described by Junglee et al. [40]. Briefly, 100 mg of liquid nitrogen-ground sample was lysed and acidified with 100 µL of 0.1%TCA, 100 µL of 10 mM potassium phosphate buffer pH 7, 100 µL of FARB lysis buffer (Favorgen, Biotech Corp, Ping-Tung, Taiwan), and 500 µL of 1M KI. Subsequently, samples were vortexed for 10 min at room temperature in darkness and centrifuged at 12,000× *g* for 15 min at 4°C. Then, 300 µL of recovered supernatant were placed in a 96-well plate and absorbance was measure at 350 nm in a microplate reader SPECTROStar Nano (BMG LABTECH, Offenburg, Germany). A standard curve using commercial H_2_O_2_ (Merck, Darmstadt, Germany) was constructed for assay calibration. Data values were normalised to protein concentrations, as determined by the Bradford method [41].

### 4.4. Determination of Lipid Peroxidation

As a proxy for lipid peroxidation, the levels of thiobarbituric acid reactive substance (TBARS) were measured according to Sáez et al. [24]. A total of 100 mg of grounded tissue were lysed with 300 µL of 0.1 % TCA, adding four glass beads (2 mm), and vortexed for 5 min at room temperature. After vortexing, samples were centrifuged at 17,800 g for 15 min at 4°C, and 200 µL of supernatant were mixed with 200 µL of 0.5 % TBA, and incubated at 95 °C for 45 min. Then, samples were cooled to room temperature and the absorbance of 200 µL of the mixture was measured at 532 nm in a microplate reader. Commercial malondialdehyde (MDA; Sigma-Aldrich, St. Louis, MO, USA) was used to construct a standard curve. Data were normalised to protein concentration, as determined by the Bradford method [41].

### 4.5. Oxidised and Reduced Glutathione

Reduced (GSH) and oxidised (GSSG) glutathione levels were determined using the enzymatic recycling method [42] as described for brown macroalgae in Sáez et al. [24], but optimised for *U. compressa*. Briefly, for total glutathione (GSH + GSSG), 100 mg of liquid nitrogen-grinded sample was lysed with 500 µL of 0.1M HCl and vortexed for 10 min at room temperature. Supernatant was recovered by centrifugation at 7400× *g* for 15 min at 4 °C, and neutralised with 500 µL of 500 mM sodium phosphate buffer pH 7.5. Then, 50 µL was mixed with 250 µL of Buffer GR containing: 100 mM sodium phosphate buffer pH 7.5, 0.1 mM EDTA, 0.3 mM NADPH, 0.6 U glutathione reductase, and 0.2 mM 5,5′-dithiobis (2-nitrobenzoic acid) (DTNB or Ellman’s reagent, Sigma-Aldrich, St. Louis, MO, USA). Immediately after the start of the reaction, absorbance at 412 nm was recorded in the microplate reader every 20 s for a total of 5 min. For GSSG quantification, 50 µL of the neutralised supernatant was pre-treated with 1 µL of 1M 4-vinylpiridine for 45 min at room temperature as a GSH masking agent, and then mixed with 250 µL of GR buffer to complete the reaction. GSH content was obtained by subtracting GSSG content from total glutathione. Standard curves using commercial GSH (Sigma-Aldrich) were constructed for assay calibration. Data were normalised to protein concentrations, determined by the Bradford method [41].

### 4.6. Oxidised and Reduced Ascorbate

Reduced ascorbate (ASC) and dehydroascorbate (DHA) were measured according to the ferric reducing/antioxidant power assay [24,43], with modifications. Briefly, 100 mg of grounded sample was lysed with 600 µL of 0.1 M HCl, vortexed for 10 min at room temperature, and centrifuged at 17,800 g for 15 min at 4 °C. Then, for ASC quantification, 10 µL of supernatant was mixed with 290 µL of FRAP solution containing: Sodium acetate buffer 300 mM pH 3.6, 20 mM FeCl_3_, and 10 mM 2,4,6-tripyridyl-s-triazine (TPTZ). Absorbance at 593 nm was immediately measured in the microplate reader. For total ascorbate (ASC + DHA) determination, 250 µL of the obtained supernatant was incubated with 2.5 µL of 100 mM dithiothreitol (DTT) for 1 h at room temperature. Then, 2.5 µL of 5% was *N*-ethylmaleimide applied to stop the reaction and 10 µL were used for the reaction with 290 µL of FRAP solution following the protocol described above. A standard curve using commercial L-ascorbate (Sigma-Aldrich) was constructed for assay calibration. Data were normalised to protein concentrations, determined by the Bradford method [41].

### 4.7. RNA Extraction

All samples were homogenised using mortars with liquid nitrogen. Then, RNA extraction was performed using 100 mg of ground tissue with the FavorPrep Plant Total RNA Purification Mini Kit (Favorgen, Biotech Corp, Ping-Tung, Taiwan), according the manufacturer’s instructions. RNA purity was determined through electrophoresis gel and spectrophometrically by 260/280 ratio, using a nanoplate within the microplate reader. RNA quantification was carried out by fluorescence using the Quant-iT RiboGreen RNA Assay Kit (Invitrogen, Waltham, MA, USA) in a QFX Fluorometer (DeNovix, Wilmington, DE, USA).

### 4.8. cDNA Synthesis and qPCR

Synthesis of cDNA was carried out with 500 ng of total RNA using the ProtoScript First Stand cDNA Synthesis Kit (New England BioLabs, Ipswich, MA, USA), according to the manufacturer’s guidelines. Then, for the qPCR reaction, 50 ng of cDNA (2 µL) were used together with 0.25 µM of each primer and the Brilliant II SYBR Green QPCR Master Mix (Agilent Technologies, Santa Clara, CA, USA) 1X (final volume of 20 µL). The qPCR program was: Initial denaturation at 95 °C for 5 min, 40 cycles of: 95 °C for 30 s, 55 °C for 30 s, 72 °C for 40 s, and final extension at 72°C for 10 min. All reactions were performed in a MIC qPCR Magnetic Induction Cycler (Bio Molecular Systems, Queensland, Australia). Primers were designed specifically for each gene using the GenScript online tool primer designer (https://www.genscript.com/tools/pcr-primers-designer) (Table 1), based on public transcriptome sequences from Rodríguez et al. [44]. The analysed transcripts corresponded to genes coding *CAT* (catalase), *SOD* (superoxide dismutase), *TRX* (thioredoxin), *APX* (ascorbate peroxidase), *DHAR* (dehydroascorbate reductase), and *GS* (glutathione synthase). Relative expression analyses were based in the 2^-ΔΔCt^ method using 18S rRNA as housekeeping gene [45].

### 4.9. Statistical Analyses

Interactive effects in all parameters content were analysed using ANOVA. This test was performed for *U. compressa* including treatment (one-way) as fixed factor for the whole dataset (mean ± SE, *n* = 3). Each time period (6 h, 24 h, 48 h, and 6 d) included 9 different factors: T1, T2, T3, T4, T5, T6, T7, T8, and T9, assessed at a 95% confidence interval. Homogeneity of variance was checked with Cochran. Student Newman Keuls tests (SNK) were conducted after observing significant interactions in the ANOVA. All data complied with the requirements of normality and homogeneity of variance. Analyses were conducted with SPSS v.21 (IBM, USA). A PCO analysis was performed to detect patterns between the parameters on the basis of an Euclidean distance using PERMANOVA+ for PRIMER6 package [46]. PCO analyses were conducted for results in vivo of H_2_O_2_, TBARS, total glutathione ascorbate, as well as for the expression of the assessed genes. Finally, a PCO was also carried out considering the data contained in this investigation plus the results in the parallel article Celis-Plá et al. [1].

## Figures and Tables

**Table 1:** Genes and primers for qPCR analyses.

Gene	Encoded Product	Primer ID	Sequence 5′-3′	Start Position	Amplicon Size (bp)
*CAT*	Catalase	F1_CAT_Ulva	AGCGGAACAAGTCGGCGGCAA	717	208
		R1_CAT_Ulva	CAGCACCATGCGGCCAACGG	905	
*SOD*	Superoxide dismutase	F1_SOD2_Ulva	CCCTGCACCGCCGTCTGTCG	22	255
		R1_SOD2_Ulva	CGCGCTGCTGATCTTCGGAGCA	255	
*TRX*	Thioredoxin	F1_Trx2_Ulva	GCTGAGTGGCTCCTCTTCGCTGCAT	3	224
		R1_Trx2_Ulva	CGGCTGTGCTCACTGGTGCGT	206	
*APX*	Ascorbate peroxidase	F1c_Apx1_Ulva	AGGACGCGTGGCCCAAGTGC	131	297
		R1c_Apx1_Ulva	GTCTCCGCCGGATGGCCAAGG	408	
*DHAR*	Dehydroascorbate reductase	F1_Dhar_Ulva	CGCGGACTCCGGCGACATCT	309	327
		R1_Dhar_Ulva	AGCGCTGCGAGCTCCTCTGG	616	
*GS*	Glutathione synthetase	F1_GS_Ulva	TGGCGGCGAAGCTGCAGGAA	1322	245
		R1_GS_Ulva	GCCTGCCGCAACACCTCCCT	1547	
*18S*	18S rRNA subunit	18S-F-1183	AAT TTG ACT CAA CAC GGG	1183	448
		18S-R-1631	TAC AAA GGG CAG GGA CG	1631	

## Supplementary Materials

Supplementary materials can be found at https://www.mdpi.com/1422-0067/20/18/4546/s1.

## Abbreviations

MAPK	mitogen activated protein kinases
ERK	Extracellular Signal Regulated Kinases
JNK	c-Jun N-terminal kinases
p38	cytokinin specific binding protein
ROS	reactive oxygen species
GSH	reduced glutathione
GSSG	oxidised glutathione
ASC	reduced ascorbate
DHA	dehydroascorbate (form of oxidised ascorbate)
CAT	catalase
SOD	superoxide dismutase
TRX	thioredoxin
APX	ascorbate peroxidase
DAHR	dehydroascorbate reductase
GS	glutathione synthase
