# Supplementary material for: MAPK Pathway under Chronic Copper Excess in Green Macroalgae (Chlorophyta): Involvement in the Regulation of Detoxification Mechanisms
Source: Int J Mol Sci. 2019 Sep 13;20(18):4546. doi: 10.3390/ijms20184546 (PMC6771120; doi:10.3390/ijms20184546)
Supplement: Supplementary file 1 [file ijms-20-04546-s001.pdf]

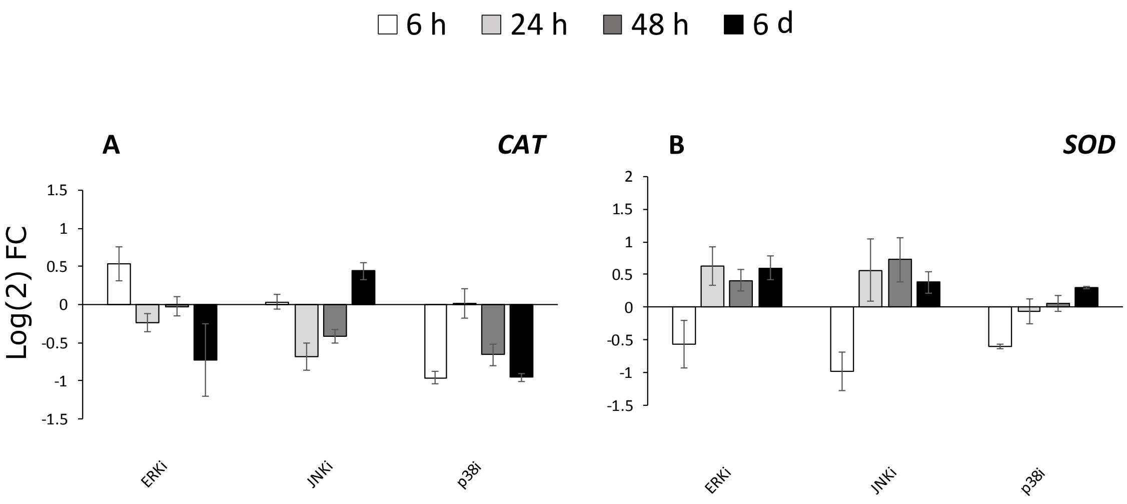


**Supplementary figure 1.** Gene expression of catalase (*CAT*; A) and superoxide dismutase (*SOD*; B) in *U. compressa* exposed to control conditions and MAPK inhibitors; levels of expression were relative to *U. compressa* under control conditions. Treatments consisted in: T1) 5 µM MAPK ERK inhibitor PD98059 in seawater (ERKi) ; T2) 5 µM MAPK JNK inhibitor SP600125 in seawater (JNKi); T3) MAPK p38 inhibitor SB203580 in seawater (p38i). Samples were analyzed after 6 h (A), 24 h (B), 48 h (C) and 6 d (D) treatments. Treatments for *SOD* and *CAT* did not present significant differences at 95% confidence interval ($p > 0.05$). Plots are represented as mean ± SE ($n = 3$).
